# USP20 competitively binds to STUB1 to enhance CTSL expression and promote epithelial‐mesenchymal transition in head and neck squamous cell carcinoma

**DOI:** 10.1002/ctm2.70520

**Published:** 2025-11-19

**Authors:** Lunhua Guo, Baihui Zhang, Xiaoqiao Cui, Xueying Wang, Jiaqing Xiao, Susheng Miao, Kaibin Song, Ji Sun

**Affiliations:** ^1^ Department of Head and Neck Surgery Harbin Medical University Cancer Hospital Harbin Heilongjiang China; ^2^ Department of Otolaryngology Head and Neck Surgery Xiangya Hospital Central South University Changsha Hunan People's Republic of China; ^3^ Institute of Heilongjiang Province Center for Disease Control Disinfection and Infection Harbin Heilongjiang People's Republic of China

**Keywords:** deubiquitinase, EMT, HNSCC, STUB1, USP20

## Abstract

**Rationale:**

Metastatic head and neck squamous cell carcinoma (mHNSCC) poses a significant threat to patient survival. Previous studies have identified cathepsin L (CTSL) as a key driver of tumourigenesis, metastasis and chemoresistance. However, the regulatory mechanisms underlying CTSL expression remain poorly understood.

**Methods:**

A specific deubiquitinase responsible for CTSL expression was identified through treatment with broad‐spectrum deubiquitinase inhibitors and mass spectrometry analysis. The colocalization of CTSL and USP20 in the cytoplasm was examined using confocal microscopy. The effects of CTSL or USP20 depletion on tumour biological behaviour were evaluated through various in vitro and in vivo assays.

**Results:**

We identified USP20 as a specific deubiquitinase of CTSL. USP20 mediates the deubiquitination and stabilization of CTSL, thereby promoting epithelial‐to‐mesenchymal transition and cancer stem cell renewal, ultimately enhancing metastatic potential and chemoresistance. Notably, USP20 competes with STUB1 for CTSL binding, further driving the malignant phenotype of HNSCC. Analysis of clinical samples revealed that both CTSL and USP20 are highly expressed in metastatic HNSCC tissues, with a positive correlation between their expression levels.

**Conclusions:**

Our study reveals a novel mechanism in which USP20 competitively interacts with STUB1 to stabilize CTSL and promote tumour progression. These findings provide preclinical evidence supporting USP20 as a potential therapeutic target for overcoming metastasis and chemotherapy resistance in HNSCC.

**Key points:**

- USP20 deubiquitinates and stabilizes CTSL.
- STUB1 promotes CTSL ubiquitination and degradation.
- USP20 competitively binds to CTSL in competition with STUB1.
- Targeting USP20 sensitizes cancer cells to cisplatin or paclitaxel.

## Graphical abstract

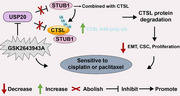

## 1 INTRODUCTION

Head and neck squamous cell carcinoma (HNSCC) is a prevalent malignancy encompassing cancers of the oral cavity, pharynx and larynx, with a global annual incidence exceeding 600 000 cases.^1^, ^2^ Despite therapeutic advances, HNSCC exhibits high rates of local recurrence and lymph node metastasis, resulting in poor overall survival (OS).^3^, ^4^ Current therapeutic modalities, including chemotherapy (e.g. cisplatin and paclitaxel), radiotherapy and limited targeted therapies, frequently yield suboptimal outcomes owing to tumour heterogeneity, chemoresistance and metastatic potential.^5^ Therefore, elucidating the molecular mechanisms underlying HNSCC progression, particularly those governing metastasis, is essential for identifying novel therapeutic targets and enhancing patient prognosis.

Cathepsin L (CTSL), a lysosomal cysteine protease, is increasingly implicated in EMT and tumour progression across multiple cancers, including HNSCC, breast and lung cancer.^6^, ^7^, ^8^, ^9^ Our previous studies found that CTSL promoted laryngeal cancer autophagy through the IL6‐JAK‐STAT3 signalling pathway.^7^ CTSL is overexpressed in tumour tissues relative to normal tissues and correlates with advanced clinical stages, lymph node metastasis and unfavourable prognosis.^10^, ^11^ Through degradation of extracellular matrix components and activation of signalling pathways, including Wnt/β‐catenin and TGF‐β, CTSL promotes tumour cell invasiveness and EMT.^12^, ^13^, ^14^ Given its pathological significance, targeting the regulatory mechanisms governing CTSL expression offers a promising strategy for attenuating HNSCC metastasis and enhancing therapeutic outcomes.

Epithelial‐mesenchymal transition (EMT) is a dynamic process through which epithelial cells lose polarity and adhesion, adopting a mesenchymal phenotype characterized by enhanced motility and invasiveness.^15^ In cancer, EMT critically drives tumour invasion, metastasis and the acquisition of cancer stem cell (CSC)‐like properties, thereby conferring therapy resistance and promoting tumour recurrence.^16^

Protein ubiquitination and deubiquitination are essential post‐translational modifications that regulate protein stability and function, thereby modulating diverse cellular processes, including tumour progression.^17^ Ubiquitin‐specific protease 20 (USP20), a deubiquitinating enzyme (DUB), is a pivotal regulator in cancer biology, stabilizing substrates that drive proliferation, metastasis and therapy resistance.^18^, ^19^ Recent studies have elucidated USP20's role in stabilizing proteins that promote oncogenic signalling, including SNAI2 in breast cancer and RETREG1 in reticulophagy, highlighting its context‐dependent functions.^20^, ^21^ In contrast, the E3 ubiquitin ligase carboxyl terminus of Hsc70‐interacting protein (STUB1) promotes protein degradation by facilitating ubiquitin conjugation, frequently counteracting DUB activity.^22^ The interplay between USP20 and STUB1 in regulating specific substrates remains largely unexplored, particularly in HNSCC.

In this study, we establish USP20 as a pivotal regulator of CTSL expression in HNSCC. We demonstrate that USP20 competitively binds STUB1, inhibiting STUB1‐mediated ubiquitination and degradation of CTSL, thus stabilizing CTSL protein levels. This stabilization promotes CTSL‐driven EMT, CSC self‐renewal and metastasis in HNSCC, while also conferring chemoresistance. Notably, silencing USP20 destabilizes CTSL, attenuates EMT and sensitizes HNSCC cells to chemotherapeutic agents, including cisplatin. Our findings delineate a novel USP20‐STUB1‐CTSL axis that drives HNSCC progression, underscoring USP20 as a promising therapeutic target for mitigating metastasis and enhancing treatment efficacy in HNSCC.

## 2 METHODS

### 2.1 Patient samples

Human tumour and adjacent normal tissue samples were collected from patients diagnosed with hypopharyngeal carcinoma and undergoing surgical resection in the Department of Head and Neck Surgery at Harbin Medical University Cancer Hospital (Heilongjiang, China). In addition, patients who received surgical treatment in the same department between January 2020 and January 2022 were followed up, and the date of death or the last follow‐up was recorded. The protocol for tissue collection was approved by the Ethics Committee of Harbin Medical University Cancer Hospital and was conducted in accordance with the principles of the Declaration of Helsinki.

### 2.2 Cell culture

Human embryonic kidney (HEK293T) cells and immortalized human HNSCC cell lines, including FaDu, TU212, TU686, LCC‐1 (Sangon Biotech), HN8 (SSRCC), TU138 (Stem recel) and DOK (Yubo Bio), were cultured in DMEM or RPMI 1640 medium (Solarbio Science & Technology Co.) supplemented with 10% foetal bovine serum (Cegrogen), 100 U/mL penicillin and 100 µg/mL streptomycin. Cells were maintained at 37°C in a humidified atmosphere with 5% CO_2_. All cell lines were periodically tested for short tandem repeat profiling and mycoplasma contamination.

### 2.3 Antibodies and regents

The antibodies used for immunoblot and immunoprecipitation were listed as follows: anti‐CTSL (27952‐1‐AP, Proteintech), anti‐USP20 (17491‐1‐AP, Proteintech), anti‐β‐tubulin (10094‐1‐AP, Proteintech), anti‐ubiquitin (ab134953, Abcam), anti‐STUB1 (68407‐1‐Ig, Proteintech), anti‐Vinmentin (#5741, CST), anti‐N‐cadherin (#13116, CST), HA Tag (#3724, CST), Myc Tag (#2276, CST) and DYKDDDDK Tag (#14793, CST).

### 2.4 Western blotting

The detailed steps are as described earlier.^23^

### 2.5 Constructs and generation of overexpression or knockout cell lines

Overexpression plasmid plasmids for USP20 and CTSL were constructed by Ubigene Bio Co., Ltd. A stop codon was inserted before the HA tag of pCMV‐C‐HA to construct pCMV‐USP20‐CA (C154S, USP20 inactive mutation). shRNAs sequences were designed to target the target genes and inserted into pGPU6/GFP/puromycin vector. Detailed information can be found in Table S1. The cell transfections were described as our previously described.^23^, ^24^

### 2.6 Wound healing assay and colony formation

HN8 and FaDu cells were cultivated within six‐well plates. Once the cells had achieved a confluence level of 95%, precise incisions were made vertically across the monolayers. Subsequently, a medium devoid of serum was employed to sustain the cells. Visual documentation was conducted, and the distances between the incisions were measured at both the initial time point (0 h) and after a lapse of 24 h.

For colony formation assays, 8000 FaDu cells and 10 000 HN8 cells were seeded into each well of 6 cm dish. After approximately 2 weeks. After removing the culture medium, complete medium containing potassium luciferin (Beyotime, #ST196‐100 mg) was added, and imaging was performed using the FMT4000 system (Perkin Elmer). Fluorescence intensity was quantified to analyse different induced differences.

### 2.7 Immunoprecipitation (IP) and mass spectrometry

Cells were collected and lysed in IP lysis buffer (NCM Biotech, P70100) supplemented with protease inhibitors (TargetMol, C0001) for 30 min on ice at 4°C. The lysates were then centrifuged at 12 000 × *g* for 15 min, and the supernatant was collected. A portion of the supernatant was reserved for Western blot analysis. The remaining lysates were incubated overnight at 4°C with gentle agitation for immunoprecipitation (IP) using the indicated antibodies. All subsequent procedures were performed according to the manufacturer's instructions. Finally, the bound proteins were eluted either by boiling or using FLAG peptide and subjected to Western blot or LC‐MS/MS analysis.

### 2.8 Immunohistochemical staining evaluation

The expressions of USP20 and CTSL on the HNSCC tissue were performed by immunohistochemical (IHC) staining. All slides were evaluated and scored by two board‐certificated pathologists in our hospital, who were blinded to clinical information. If a disagreement occurred, the slides were re‐examined to achieve a final consensus. The detailed steps are as described earlier.^23^

### 2.9 Opal multiplex immuno‐histochemistry

The detailed steps are as described earlier.^23^

### 2.10 Mouse tumour model and treatment

The animal study protocol was approved by the Institutional Review Board of Harbin Medical University Cancer Hospital. Male nude mice were purchased from Vital River Laboratory Animal Technology Co., Ltd., and housed in the Department of Animal Science, Harbin Medical University. FaDu cells subjected to different treatments were subcutaneously injected into the dorsal region of each mouse. Mice were administered either saline or cisplatin (2 mg/kg) every 2 days until euthanasia. At the end of the experiment, mice were euthanized, and tumours were excised and weighed. For lung metastasis studies, treated FaDu cells were injected into the tail veins of 6‐week‐old nude mice in two separate doses to avoid embolism‐related mortality caused by a single large bolus of tumour cells and to ensure sufficient tumour burden. After 4 weeks, the mice were euthanized. Prior to euthanasia, tumour images were captured using a small animal imaging system (FMT400010, PerkinElmer), and the data were analysed using Living Image software version 4.4 (PerkinElmer).

### 2.11 Confocal microscopy and immunofluorescence

The detailed steps are as described earlier.^23^

### 2.12 IC50

IC50 values were calculated in GraphPad Prism 10.3 using a four‐parameter logistic, variable‐slope model. Drug concentrations were converted to µM, log10‐transformed and fitted without constraining top or bottom plateaus. LogIC50 estimates were back‑transformed to µM with 95% confidence intervals (CIs) obtained by Prism's profile‑likelihood routine. Curve fits were accepted when *R* ^2^ ≥ .99 and residuals were randomly distributed. IC50 from at least three independent experiments are reported as mean  ±  SD; group differences were tested on logIC50 values with one‐way ANOVA followed by Tukey's post‐hoc test (two‐tailed *p* < .05).

### 2.13 Software and statistical analysis

Statistical analyses were performed using Microsoft Excel, GraphPad Prism and R (version 4.5.0). Data are presented as mean ± SD from three independent experiments. Differences between two groups were analysed using either a two‐sample *t*‐test or an exact Wilcoxon rank‐sum test, depending on data normality. For comparisons among more than two groups, one‐way ANOVA was applied when assumptions of normality and homogeneity of variance were satisfied; otherwise, the Kruskal–Wallis test was used. Univariate and multivariate Cox proportional hazards regression analyses were performed to evaluate the association between potential prognostic factors and OS. Variables with *p* < .05 in univariate analysis were included in the multivariate model. The proportional hazards assumption was assessed using Schoenfeld residuals. Hazard ratios and corresponding 95% CIs were calculated. A two‐tailed *p* ≤ .05 was considered statistically significant (*p < .05; **p < .01; ***p < .001; ****p < .0001).

For limiting dilution analysis, parameters were estimated using a generalized linear model with a complementary log‐log link. The generalized Pearson chi‐square was used to assess the goodness‐of‐fit to the model. The analyses were done using programme L‐Calc and ELDA online software.^25^

## 3 RESULTS

### 3.1 CTSL promotes HNSCCs migration and invasion

**FIGURE 1:**
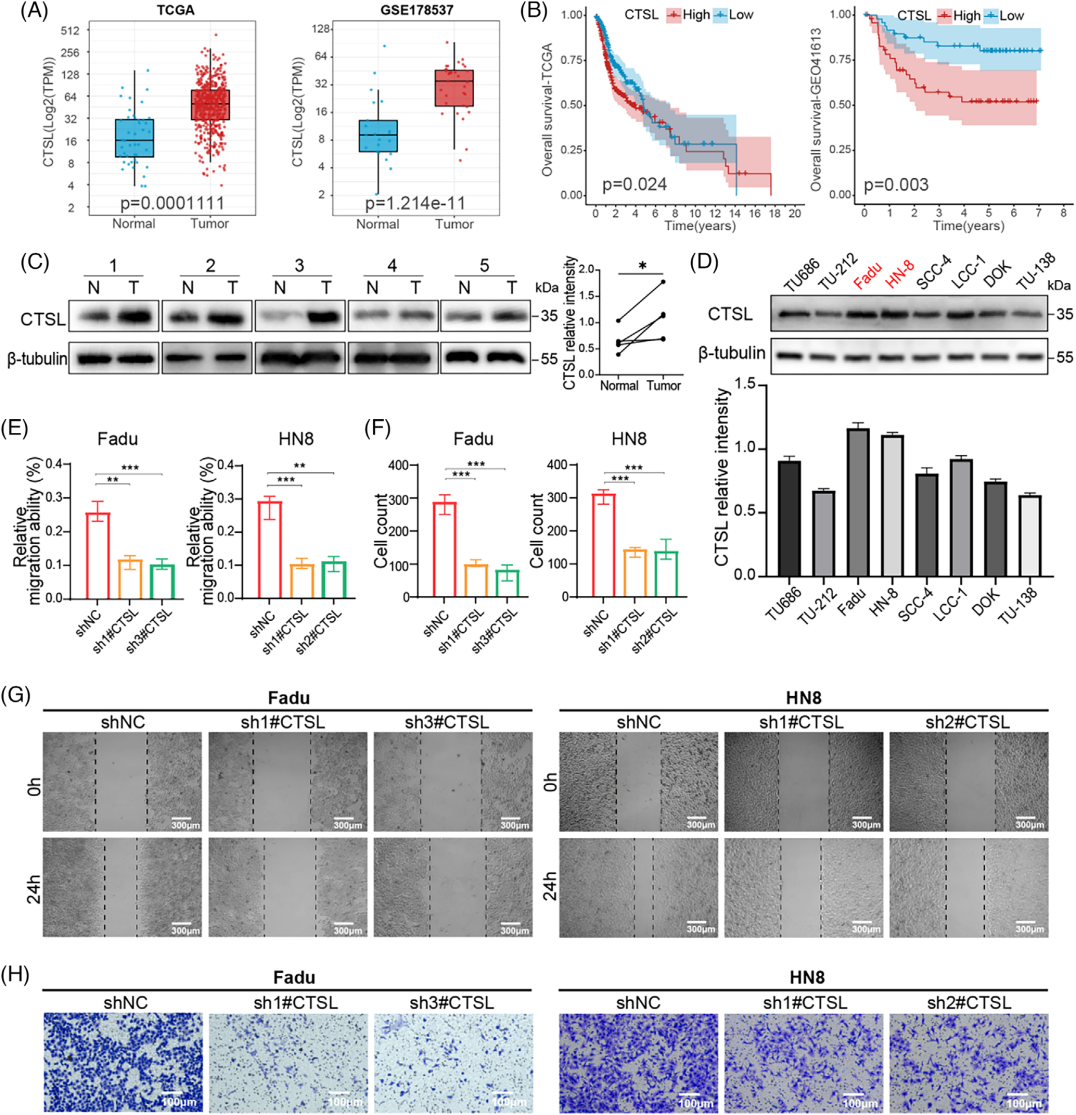
Cathepsin L (CTSL) expression is significantly upregulated during head and neck squamous cell carcinoma (HNSCC) development. (A) CTSL level in HNSCC tumour samples and normal controls from TCGA and GEO data. Data are presented as median and interquartile range (IQR). Statistical differences among groups were assessed using *t*‐test. *p* < .05 was considered statistically significant. (B) Kaplan–Meier curve depicting the overall survival of HNSCC patients from TCGA and GEO database. Survival curves were estimated using the Kaplan–Meier method and compared using the log‐rank test. (C) Western blot analysis of CTSL protein expression in paired normal (N) and tumour (T) tissues from five patients. Differences were analysed using a paired *t*‐test. (D) Western blot analysis of CTSL protein expression in various tumour cell lines. Data are presented as mean ± SD (*n* = 3). (E–H) Wound healing and trans‐well migration assays in FaDu and HN8 cells transfected with shNC or shCTSL. (E) Quantification of relative wound healing percentage (%) in FaDu and HN8 cells. (F) Quantification of migrated FaDu and HN8 cells. Data are presented as mean ± SD (*n* = 3). Statistical significance was determined using a two‐tailed unpaired *t*‐test. (G) Representative images of scratch wounds at 0 h and 24 h. Scale bar: 300 µm. (H) Representative images of trans‐well migration assay. Scale bar: 100 µm. *p < .05, **p < .01, ***p < .001.

To investigate the expression pattern of CTSL in HNSCC, we first analysed its mRNA levels in tumour versus normal tissues using TCGA and GSE178537 datasets. CTSL was significantly upregulated in tumour tissues compared with normal tissues in both datasets (Figure 1A). Consistently, Kaplan–Meier survival analysis demonstrated that higher CTSL expression was associated with poorer OS in HNSCC patients from both cohorts (Figure 1B), indicating a potential oncogenic role of CTSL in HNSCC. To further validate the upregulation of CTSL at the protein level, we performed Western blot analysis on five paired HNSCC tumour and adjacent normal tissues. CTSL expression was markedly elevated in tumour samples compared with their matched normal controls (Figure 1C). Additionally, CTSL protein levels were found to be highly expressed in multiple HNSCC cell lines compared to immortalized oral epithelial cells (Figure 1D), among which FaDu and HN8 cells exhibited relatively high expression and were selected for subsequent functional assays. Further, we generated CTSL knockdown cell lines using three independent shRNAs in FaDu and HN8 cells (Figure S1A,B). Wound healing assays revealed that CTSL depletion significantly impaired the migratory ability of both FaDu and HN8 cells at 24 h compared to the control group (Figure 1E,G). Moreover, trans‐well invasion assays showed that CTSL knockdown substantially suppressed the invasive capabilities of FaDu and HN8 cells (Figure 1F,H). In our previous study, we found that CTSL can activate the PI3K/AKT pathway to promote immune evasion in HNSCC.^26^ This prompted us to further examine changes in EMT. Under different conditions (treatment with Sc‐79 and MK2206), we observed that both overexpression (OE‐CTSL) and knockdown (shCTSL) of CTSL significantly affected the activity of the PI3K‐AKT pathway, particularly the level of p‐AKT (Figure S1C,D). These data suggest that CTSL may regulate the PI3K pathway upstream, thereby activating AKT signalling and ultimately influencing tumour cell proliferation and invasion.

### 3.2 USP20 interacts with and stabilizes CTSL in HNSCC cells

**FIGURE 2:**
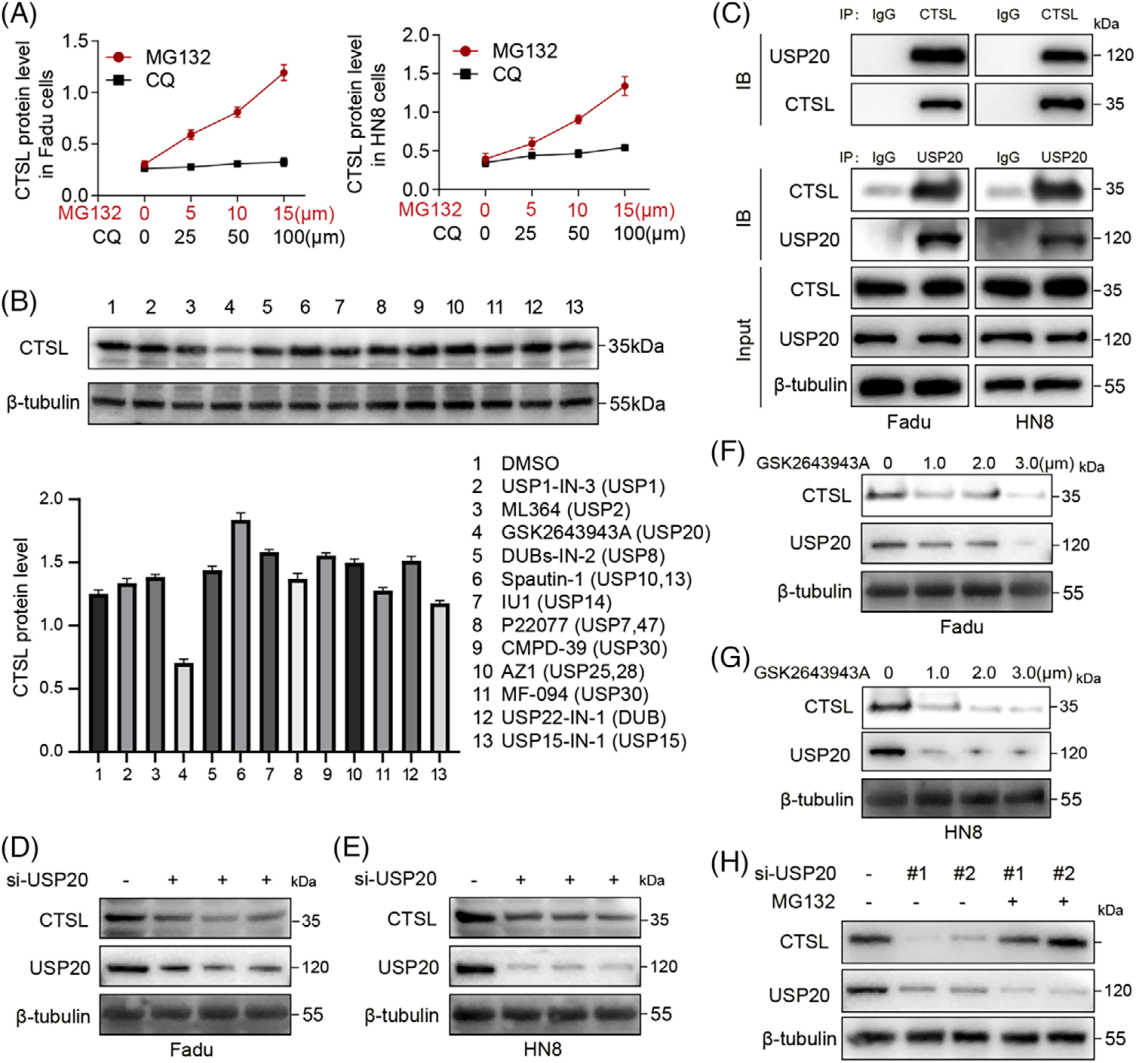
USP20 inhibition elevates cathepsin L (CTSL) protein abundance in head and neck squamous cell carcinoma (HNSCC) cells. (A) FaDu and HN8 cell lines were treated with MG132 and CQ for 24 h using concentration gradients respectively, to assess the response of CTSL protein expression levels to different treatments. Data are presented as mean ± SD (*n* = 3). (B) Western blot analysis of whole‐cell lysates (WCL) derived from FaDu cells treated with indicated inhibitors or dimethyl sulfoxide (DMSO). Data are presented as mean ± SD (*n* = 3). (C) Interaction of endogenous CTSL and USP20 in FaDu and HN8 cells was detected by co‐immunoprecipitation. (D, E) Western blot analysis showing the effect of USP20 knockdown (si‐USP20) on CTSL protein levels in FaDu and HN8 cells. (F, G) FaDu and HN8 cells were treated with different concentrations of GSK2643943A (an inhibitor of USP20) for 24 h, and then western blotting was used to detect the protein levels of CTSL and USP20. (H) FaDu cells transfected with siRNAs were treated with MG132 (20 µM) for 10 h before harvesting, and CTSL protein levels were detected by western blotting.

To explore the mechanism underlying CTSL accumulation in HNSCC, we treated FaDu and HN8 cells with the proteasome inhibitor MG132 or the lysosomal inhibitor chloroquine (CQ). CTSL protein levels were markedly increased in response to MG132 in a dose‐dependent (Figure 2A) and time‐dependent (Figure S1C,D) manner, while CQ had only a mild effect, suggesting that CTSL is primarily degraded via the ubiquitin‐proteasome pathway. To identify potential DUBs that may regulate CTSL stability, we screened a panel of DUB inhibitors and found that the USP20‐specific inhibitor GSK2643943A notably decreased CTSL protein levels (Figure 2B). Further, we employed affinity purification and mass spectrometry to interrogate CTSL interactome. Mass spectrometry analysis of protein complexes associated with Flag‐tagged overexpressed CTSL revealed interactions with multiple deubiquitinases (DUBs), including USP20, USP14, USP12, USP36, POH1, USP8, PRPF8 and USP15, of which USP20 had the highest score, implicating USP20 in CTSL regulation (Figure S1E,F). Co‐immunoprecipitation (co‐IP) assays demonstrated a robust interaction between endogenous USP20 and CTSL in both FaDu and HN8 cells, rather than USP14 and USP12 (Figure 2C and Figure S1G,H). We next investigated which domains of USP20 participate in the interaction with CTSL. USP20 contains three important domains: Zf‐UBP, UCH and DUSP domains. Three truncated mutants of USP20 were constructed: Flag‐USP20‐Zf‐UBP, Flag‐USP20‐UCH and Flag‐USP20‐DUSP (Figure S1J), and transiently transfected into 293T cells along with full‐length CTSL (Flag‐CTSL). Co‐immunoprecipitation assays revealed that the USP20‐UCH domain (amino acids 145–647) binds to CTSL (Figure S1K).

To evaluate the functional relevance of USP20 in the regulation of CTSL, we used three independent siRNAs to knock down USP20 in FaDu and HN8 cells, which led to a significant reduction in CTSL protein levels (Figure 2D,E), whereas the mRNA levels showed no obvious changes (Figure S1K). Treatment with GSK2643943A also led to dose‐dependent downregulation of CTSL in both cell lines (Figure 2F,G). Importantly, the MG132‐mediated rescue of CTSL levels in USP20‐silenced cells further confirmed that USP20 maintains CTSL stability by protecting it from proteasomal degradation (Figure 2H). Collectively, these results indicate that USP20 directly interacts with and stabilizes CTSL in HNSCC cells by antagonizing its proteasomal degradation.

### 3.3 USP20 stabilizes CTSL by removing K48‐linked polyubiquitin chains

**FIGURE 3:**
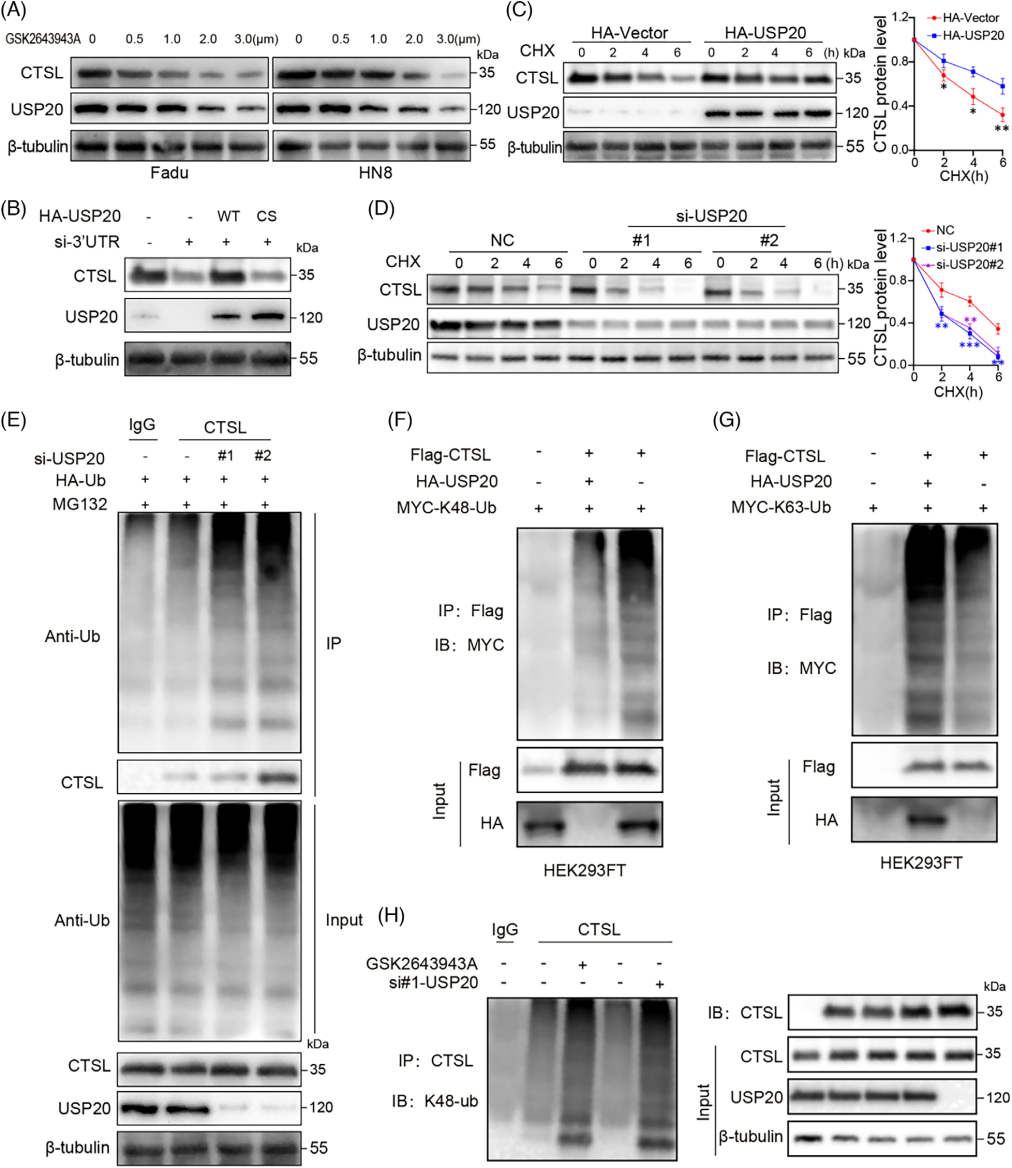
USP20 regulates cathepsin L (CTSL) stability through deubiquitination. (A) Western blot analysis of CTSL and USP20 expression levels in FaDu and HN8 cells treated with different concentrations of the USP20 inhibitor GSK2643943A for 24 h. (B) FaDu cells stably expressing HA‐vector, HA‐USP20 or HA‐USP20‐CS were transfected with USP20 3′ UTR siRNA, and the indicated antibodies were used for the western blotting. (C, D) Cycloheximide chase assay analysing the degradation rate of CTSL following USP20 overexpression (C) or knockdown (D) in FaDu cells. Line graph depicting the percentage of remaining CTSL protein levels over time. Data are presented as mean ± SD (*n* = 3). Statistical significance was assessed using a t‐test (C) or ANOVA (D). *p < .05, **p < .01, ***p < .001. (E) Ubiquitination assay showing the effect of USP20 knockdown on the ubiquitination level of endogenous CTSL in FaDu cells treated with MG132. (F, G) Co‐transfection experiments in HEK293FT cells demonstrating that USP20 preferentially reduces K48‐linked (F) but not K63‐linked (G) ubiquitination of CTSL. (H) Immunoprecipitation assay confirming that both USP20 knockdown and GSK2643943A treatment increase K48‐linked ubiquitination of endogenous CTSL in FaDu cells.

To further verify the regulatory role of USP20 in CTSL stability, FaDu and HN8 cells were treated with increasing concentrations of the USP20 inhibitor GSK2643943A. CTSL protein levels were reduced in a dose‐dependent manner, consistent with USP20 inhibition (Figure 3A). Overexpression of USP20 wild‐type but not the C154S (USP20 inactive mutation) could rescue the reduction of CTSL (Figure 3B), confirming the specificity and catalytic dependency of USP20 in CTSL regulation. CHX chase assays demonstrated that USP20 knockdown significantly reduced CTSL protein stability (Figure 3D). Conversely, ectopic expression of USP20 prolonged CTSL protein half‐life in CHX‐treated cells (Figure 3C), supporting its role in CTSL stabilization. Analysis of CTSL mRNA levels in USP20‐knockdown cells revealed that silencing USP20 had no appreciable effect on CTSL transcription (Figure S1G). To determine whether USP20 modulates CTSL ubiquitination, we performed co‐immunoprecipitation followed by anti‐ubiquitin blotting. USP20 knockdown led to a marked increase in total ubiquitinated CTSL (Figure 3E). Further analysis revealed that USP20 specifically removed K48‐linked polyubiquitin chains from CTSL (Figure 3F), but had little effect on K63‐linked ubiquitination (Figure 3G), suggesting that USP20 prevents CTSL proteasomal degradation via deubiquitination of K48‐linked chains. Finally, inhibition or knockdown of USP20 increased the K48‐linked ubiquitination of CTSL, further confirming USP20's deubiquitinating function in this axis (Figure 3H). Together, these findings indicate that USP20 stabilizes CTSL protein by selectively removing K48‐linked ubiquitin chains, thereby preventing its proteasomal degradation.

### 3.4 Diclofenac promotes CTSL degradation by disrupting USP20‐mediated deubiquitination

Given that Diclofenac has been previously reported by our group and others as an anti‐tumour agent in HNSCC,^23^, ^27^ we investigated its potential role in regulating CTSL stability. We first confirmed that Diclofenac does not regulate CTSL expression by interfering with the levels of USP20 (Figure S2A). Furthermore, we found that Diclofenac treatment significantly reduced the CTSL protein levels in HN8 and FaDu cells (Figure S2B). The proteasome inhibitor MG132 rescued this reduction, suggesting that Diclofenac promotes CTSL degradation via the ubiquitin‐proteasome system. Further, co‐treatment with CHX and Diclofenac accelerated CTSL degradation compared to CHX alone (Figure S2C), indicating that Diclofenac decreases CTSL protein stability. Consistent with this finding, immunoprecipitation of CTSL followed by anti‐HA‐Ub blotting revealed that Diclofenac significantly increased the polyubiquitination of CTSL in a time‐dependent manner (Figure S2D), further confirming enhanced proteasomal targeting of CTSL. Next, we examined whether Diclofenac interferes with the USP20‐CTSL axis. Overexpression of USP20 delayed Diclofenac‐induced CTSL degradation (Figure S2E), whereas overexpression of a catalytically inactive USP20 mutant (CS) failed to delay CTSL degradation (Figure S2F), indicating that Diclofenac antagonizes USP20 deubiquitinase activity. Moreover, knockdown of USP20 abolished Diclofenac's effect on CTSL degradation, further supporting that Diclofenac acts through inhibiting USP20‐dependent deubiquitination (Figure S2G). Collectively, these data suggest that Diclofenac promotes CTSL degradation by impairing USP20‐mediated deubiquitination, thereby enhancing proteasomal clearance of CTSL. This finding provides mechanistic insight into the anti‐tumour activity of Diclofenac in HNSCC.

### 3.5 USP20 suppressed CTSL ubiquitination by competing with STUB1

**FIGURE 4:**
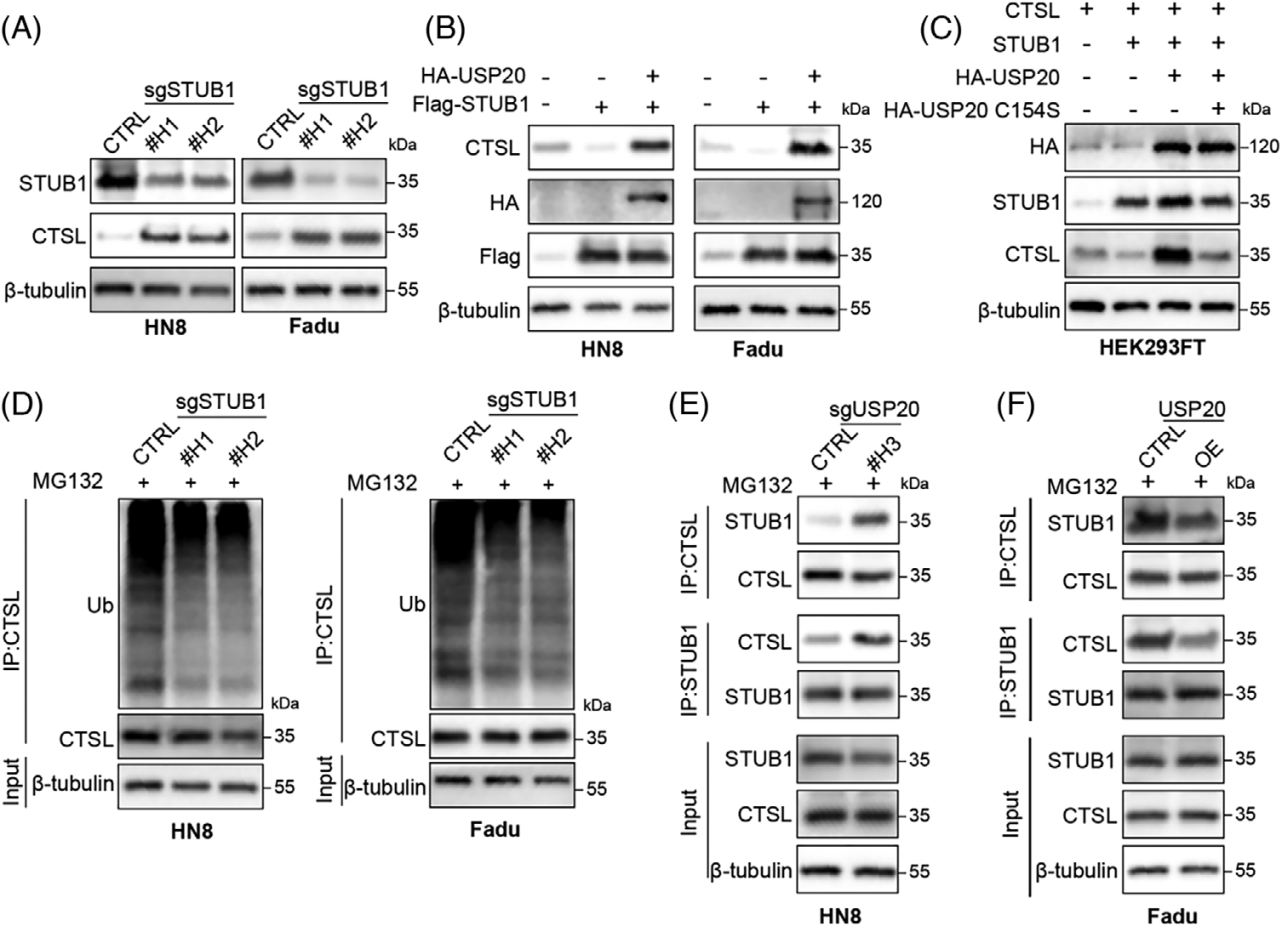
USP20 inhibits the ubiquitination of cathepsin L (CTSL) by blocking the interaction between CTSL and STUB1. (A) Western blot analysis of STUB1 and CTSL expression in HN8 and FaDu cells following knockout of STUB1. (B) Co‐IP assays in HN8 and FaDu cells transfected with Flag‐STUB1 and HA‐USP20, showing the interaction between STUB1 and CTSL. (C) HEK293FT cells were transfected with CTSL, STUB1 and HA‐tagged USP20 (WT or C154S) and analysed by Western blotting. (D) Ubiquitinylation of CTSL in HN8 and FaDu cells following STUB1 knockout, ubiquitin conjugation was assessed via Co‐IP with CTSL. (E, F) Total lysates of HN8 cells with or without USP20 knockdown (E), or FaDu cells with or without USP20 overexpression (F), were subjected to immunoprecipitation (IP) with anti‐CTSL or anti‐STUB1 antibodies, followed by Western blotting using the indicated antibodies. The cells were treated with MG132 to inhibit the proteasome.

Previous studies have shown that CTSL can be ubiquitinized and degraded by STUB1, an E3 ubiquitin ligase.^28^, ^29^ We evaluated the functional significance of STUB1 in the ubiquitinization and degradation of CTSL in HNSCC. Knockout of STUB1 using two independent sgRNAs led to a marked increase in CTSL protein levels in HNSCC cell lines HN8 and FaDu (Figure 4A), whereas CTSL mRNA levels remained unchanged (Figure S2H), indicating that STUB1 negatively regulates CTSL at the post‐transcriptional level. Here, we respectively established the HN8 and Fadu cell lines with USP20 deficiency and overexpression (Figure S2I,J). Overexpression of USP20 elevated CTSL levels, whereas co‐expression of STUB1 suppressed this effect (Figures 3B and 4B); however, the suppression was partially reversed by USP20 co‐expression, suggesting that USP20 counteracts STUB1‐mediated CTSL degradation (Figure 4B). Importantly, a catalytically inactive mutant of USP20 (C154S) failed to restore CTSL expression in the presence of STUB1, indicating that USP20's deubiquitinating activity is required for this regulation (Figure 4C). Furthermore, immunoprecipitation assays revealed that STUB1 knockdown decreased CTSL ubiquitination, confirming its role in targeting CTSL for proteasomal degradation (Figure 4D). Conversely, USP20 depletion enhanced the interaction between STUB1 and CTSL, while USP20 overexpression disrupted this interaction (Figure 4E,F). Collectively, these data demonstrate that USP20 stabilizes CTSL by competitively binding STUB1 and suppressing STUB1‐mediated CTSL ubiquitination.

### 3.6 USP20 deficiency attenuates EMT and stemness via downregulating CTSL expression

**FIGURE 5:**
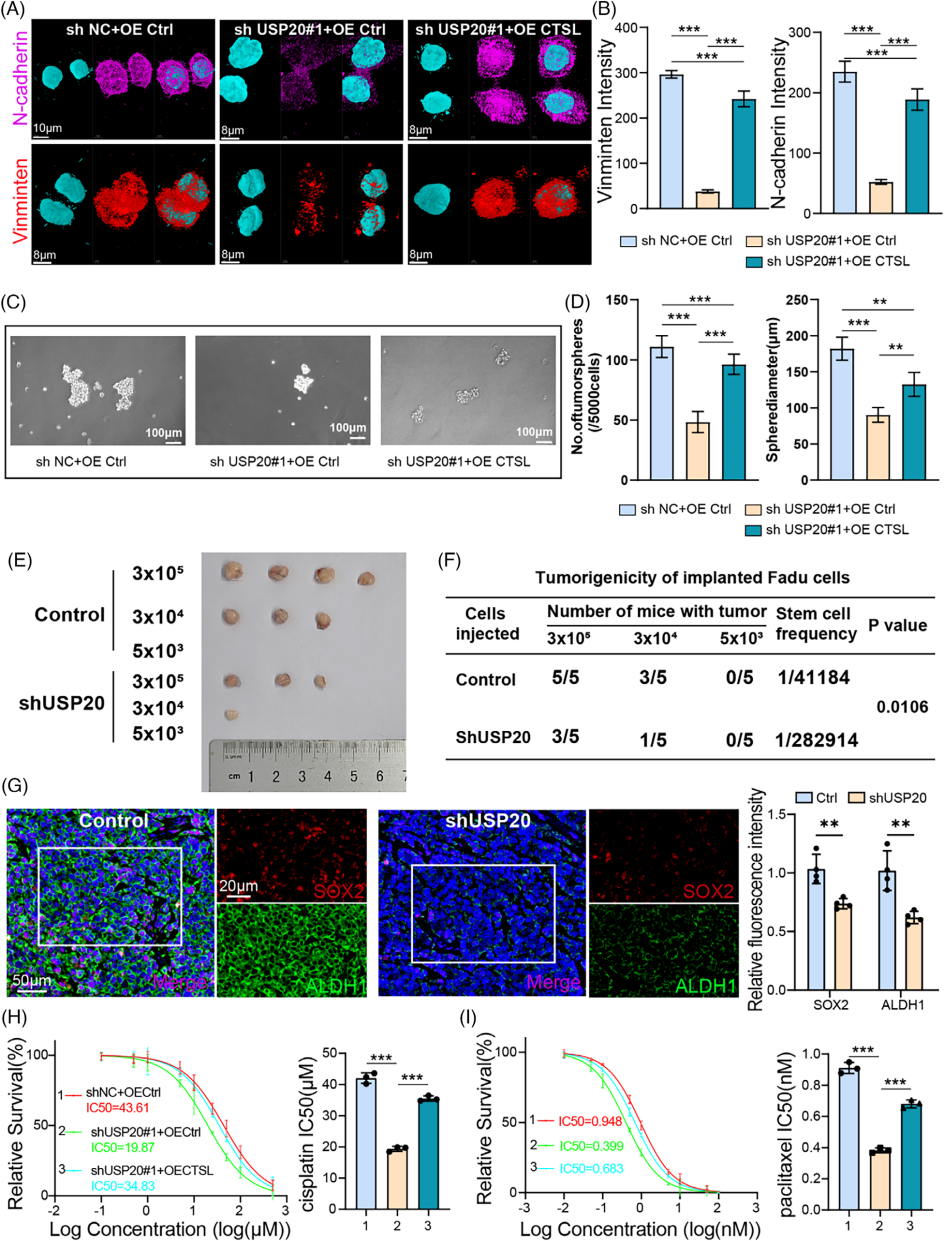
Effects of USP20 expression on cell behaviour and tumourigenicity. (A) Immunofluorescence staining showing vimentin (red) and N‐cadherin (violet) expression in cells under different conditions. (B) Quantification of vimentin and N‐cadherin fluorescence intensity in cells from panel A. Data are presented as mean ± SD (*n* = 3), statistical significance was assessed using ANOVA. (C) Bright‐field images of tumourspheres formed under different conditions. (D) Quantification of the number and diameter of tumourspheres from panel C. Data are presented as mean ± SD (*n* = 3), statistical significance was assessed using ANOVA. (E) Tumour formation assay with varying numbers of cells (5 × 10^3^–3 × 10^5^) under control and sgUSP20 conditions. (F) Table summarizing the tumourigenicity of implanted HN8 cells in mice. The number of mice with tumours and stem cell frequency are shown for control and sgUSP20 conditions. (G) Immunofluorescence staining of SOX2 (red) and ALDH1 (green) in tumour sections from control and sgUSP20 groups. Bar graph shows quantification of fluorescence intensity. Data are presented as mean ± SD (*n* = 4). Statistical significance was assessed using *t*‐test. (H, I) Cells as in panel A were treated with cisplatin (H) or paclitaxel (I) and cell survival was determined. Data are presented as mean ± SD (*n* = 3). Statistical significance was assessed using ANOVA. **p < .01, ***p < .001.

CTSL is significantly implicated in EMT, CSC self‐renewal, metastasis and chemotherapy resistance.^9^, ^30^, ^31^ We hypothesize that USP20 may promote these malignant processes by stabilizing CTSL. As shown in Figure 5A, silencing USP20 significantly reduced the expression of mesenchymal marker vimentin and N‐cadherin in comparison to negative controls. Restoration of CTSL expression reversed these alterations, indicating that USP20 promotes EMT in a CTSL‐dependent manner (Figure 5A,B). Next, we assessed the role of USP20 in cancer stemness. Sphere formation assays demonstrated that USP20 knockdown significantly reduced both the number and diameter of tumourspheres, and these effects were rescued by CTSL overexpression (Figure 5C,D). Consistently, western blot analysis showed that USP20 silencing downregulated vimentin and N‐cadherin, and upregulated E‐cadherin, further confirming EMT suppression upon USP20 depletion (Figure S3A). Moreover, the wound healing (Figure S3B) and proliferation assay (Figure S3C) revealed that USP20 knockdown significantly impaired migratory and proliferation capacity of FaDu cells, whereas CTSL overexpression restored it.

To evaluate the impact of USP20 on tumour‐initiating ability, limiting dilution xenograft experiments were performed using FaDu cells. As shown in Figure 5E,F, USP20 knockdown markedly decreased tumour formation rates and stem cell frequency. Immunofluorescence staining further confirmed decreased expression of stemness markers SOX2 and ALDH1 in USP20‐silenced tumours compared to controls (Figure 5G). EMT and CSCs play an important role in cancer metastasis and chemotherapy resistance. Drug sensitivity assays revealed that USP20 depletion enhanced sensitivity to cisplatin and paclitaxel. The IC50 values of cisplatin and paclitaxel were markedly lower in USP20‐deficient cells compared to controls, and re‐expression of CTSL reversed the increased drug sensitivity (Figure 5H,I). Collectively, these findings demonstrate that USP20 promotes EMT, migration, drug resistance and cancer stemness through upregulating CTSL expression, highlighting its potential as a therapeutic target in HNSCC.

### 3.7 USP20 promotes lung metastasis and EMT in vivo through CTSL regulation

**FIGURE 6:**
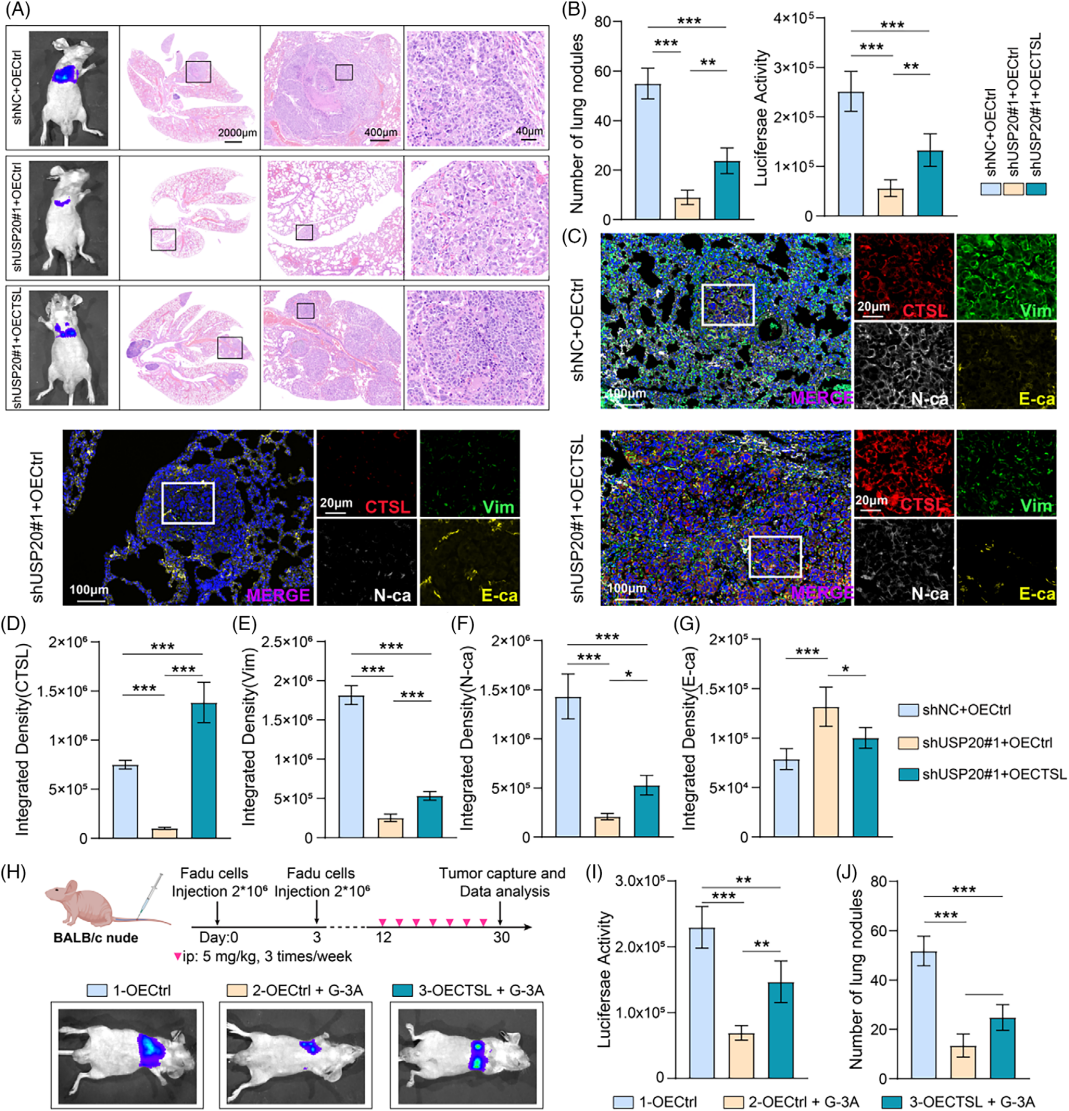
USP20 promotes tumour lung metastasis through CTSL. (A) Representative bioluminescent imaging and H&E staining of lung tissues from nude mice injected with indicated FaDu cells. Insets show magnified views of metastatic nodules. (B) Quantification of lung metastatic nodules (left) and luciferase activity (right) in the respective groups. Data are presented as mean ± SD (*n* = 5). Statistical significance was assessed using ANOVA. (C) Representative immunofluorescence staining images showing the expression of CTSL, vimentin (Vim), N‐cadherin (N‐ca) and E‐cadherin (E‐ca) in lung metastatic tissues from indicated groups. (D–G) Quantification of integrated fluorescence density for CTSL (D), vimentin (E), N‐cadherin (F) and E‐cadherin (G) in lung metastatic lesions. Data are presented as mean ± SD (*n* = 3). Statistical significance was assessed using ANOVA. (H) Schematic of experimental design for drug treatment in vivo. BALB/c nude mice were injected with FaDu cells followed by intraperitoneal treatment, and imaged 30 days later. (I, J) Quantification of luciferase activity (I) and number of lung metastatic nodules (J) in different treatment groups. Data are presented as mean ± SD (*n* = 5). Statistical significance was assessed using ANOVA. *p < .05, **p < .01, ***p < .001.

To validate the in vivo relevance of USP20 in promoting metastasis, a tail vein injection model was established using FaDu cells. As shown in Figure 6A, mice injected with sgUSP20 cells exhibited markedly fewer and smaller metastatic nodules in the lungs compared to controls, while CTSL overexpression significantly restored the metastatic burden (Figure 6B). Immunofluorescence staining of lung tissues further revealed that USP20 silencing led to reduced expression of CTSL and vimentin, and increased E‐cadherin expression, while N‐cadherin expression was also suppressed (Figure 6C). These changes were reversed upon CTSL overexpression (Figure 6D–G).

To further assess the therapeutic potential of targeting the USP20‐CTSL axis, mice inoculated with tumour cells via tail vein injection were treated with the small‐molecule USP20 inhibitor G‐3A. As illustrated in the experimental scheme (Figure 6H), compared with the control group, G‐3A significantly reduced the frequency and size of lung metastases, whereas CTSL overexpression partially restored the metastatic burden (Figure 6I,J). These in vivo results further suggest that USP20 plays a key role in promoting head and neck tumour progression by stabilizing CTSL. These results collectively demonstrate that USP20 enhances lung metastasis and EMT in vivo by upregulating CTSL expression, and that pharmacologic inhibition of USP20 may serve as a promising therapeutic strategy for CTSL‐driven cancers.

### 3.8 USP20 and CTSL are positively correlated with metastasis and poor prognosis in HNSCC

**FIGURE 7:**
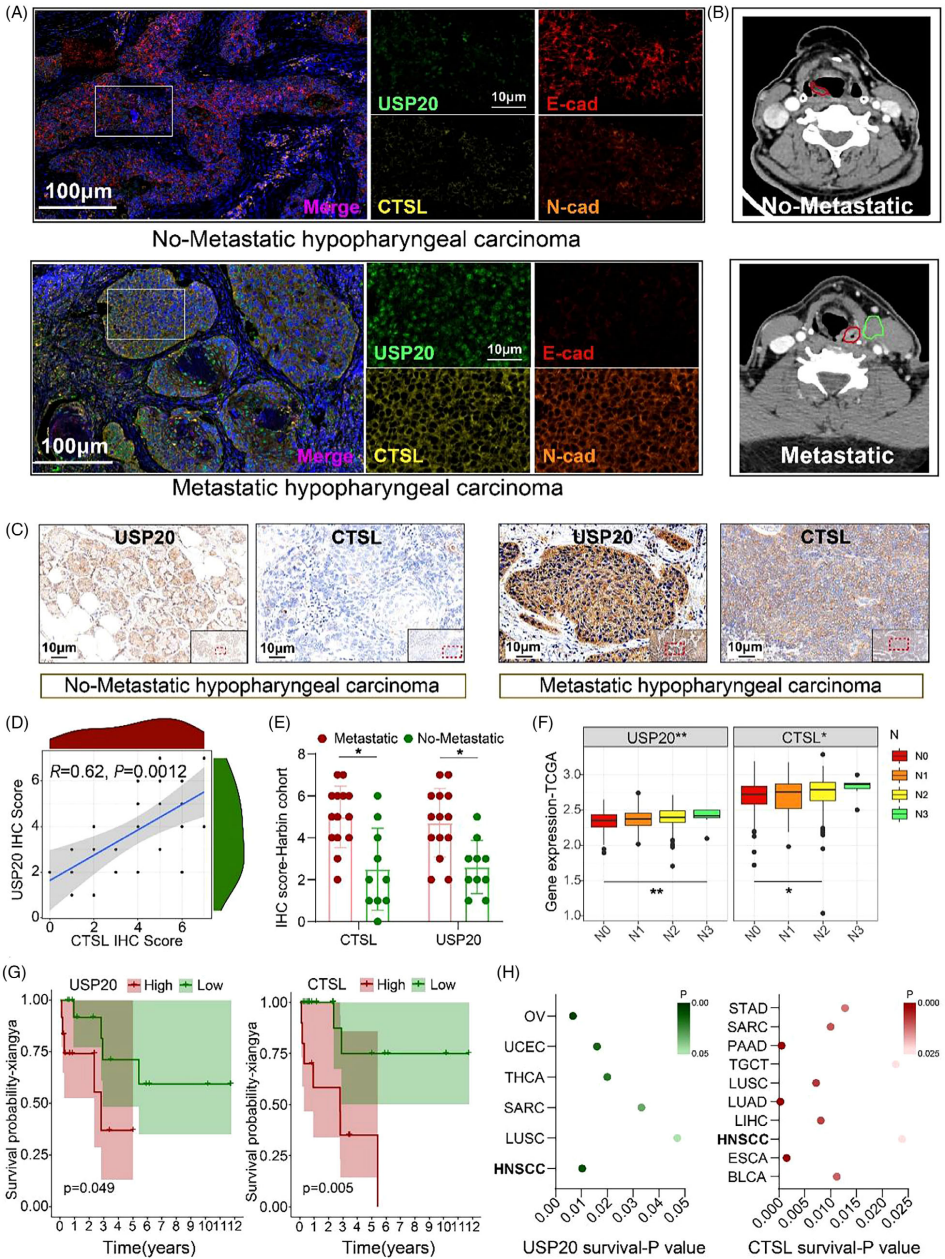
USP20 and cathepsin L (CTSL) are positively correlated with metastasis and poor prognosis in hypopharyngeal carcinoma. (A) Representative immunofluorescence staining of USP20, CTSL, N‐cad and E‐cad in non‐metastatic and metastatic hypopharyngeal carcinoma tissues. (B) Representative CT imaging showing lymph node status in non‐metastatic and metastatic hypopharyngeal carcinoma patients. Red represents the primary lesion and green represents the metastatic lesion. (C) Representative immunohistochemistry (IHC) staining of USP20 and CTSL in non‐metastatic and metastatic hypopharyngeal carcinoma tissues. (D) Correlation between USP20 and CTSL IHC scores in the hypopharyngeal carcinoma cohort (*n* = 24) was analysed using Spearman's rank correlation coefficient. (E) Quantification of USP20 and CTSL IHC scores in metastatic (*n* = 14) versus non‐metastatic (*n* = 10) hypopharyngeal carcinoma tissues from the Harbin cohort. Data are presented as mean ± SD. Statistical significance was assessed using a *t*‐test. (F) TCGA analysis of USP20 and CTSL mRNA expression across different N stages (N0–N3) in HNSCC patients. Data are presented as median and interquartile range (IQR). Statistical differences among stages were assessed using ANOVA. (G) Kaplan–Meier survival analysis comparing overall survival between high and low USP20 or CTSL expression groups in the hypopharyngeal carcinoma cohort. (H) Pan‐cancer analysis of the association between USP20 and CTSL expression and patient survival based on TCGA datasets. Shading intensity represents the magnitude of the *p* value, with darker colours indicating smaller *p* values. Survival curves were estimated using the Kaplan–Meier method and compared using the log‐rank test. *p < .05, **p < .01.

To explore the clinical significance of USP20 and CTSL in hypopharyngeal carcinoma, we first performed immunofluorescence staining on human tumour tissues, focusing on hypopharyngeal carcinoma given its dismal prognosis, therapeutic challenges and the availability of a continuously followed patient cohort in our centre. Compared with non‐metastatic tumours, metastatic hypopharyngeal carcinoma tissues exhibited significantly higher levels of USP20 and CTSL, accompanied by elevated N‐cadherin and decreased E‐cadherin expression, indicative of an enhanced EMT phenotype (Figure 7A). Corresponding CT scans further confirmed the presence of lymph node metastasis in metastatic cases (Figure 7B). IHC staining validated these observations, demonstrating higher expression levels of USP20 and CTSL in metastatic tissues compared to non‐metastatic ones (Figure 7C). Correlation analysis revealed a strong positive relationship between USP20 and CTSL expression in hypopharyngeal carcinoma samples (*R* = .62, *p* = .0012) (Figure 7D). Consistently, IHC scores of both USP20 and CTSL were significantly elevated in metastatic tumours relative to non‐metastatic tumours in the Harbin cohort (Figure 7E). Analysis of TCGA datasets showed that the expression of USP20 and CTSL progressively increased with the extent of lymph node metastasis (N stage), further supporting their association with metastatic potential (Figure 7F). Moreover, USP20 overexpression was positively correlated with lymph node metastasis and advanced AJCC stage (Table S2). Kaplan–Meier survival analyses indicated that patients with high USP20 or CTSL expression had significantly poorer OS compared to those with low expression (Figure 7G). Pan‐cancer analysis across TCGA datasets revealed that USP20 and CTSL expression were particularly associated with unfavourable prognosis in HNSCC, among other cancer types (Figure 7H). Cox proportional hazards analysis revealed that HNSCC patients with elevated CTSL expression had significantly shorter OS, indicating that higher CTSL levels serve as an independent risk factor for poor prognosis in HNSCC patients (Tables S3 and S4). Together, these findings suggest that USP20 and CTSL are closely linked to metastatic progression and poor clinical outcomes in hypopharyngeal carcinoma, highlighting their potential as prognostic biomarkers and therapeutic targets.

## 4 DISCUSSION

HNSCC is the sixth most common cancer worldwide. Its primary risk factors include heavy tobacco use and alcohol consumption. However, despite a declining incidence of these behaviours, the overall incidence of HNSCC continues to rise. Notably, patients often fail to detect the disease at an early stage, with most diagnoses occurring at locally advanced stages. HNSCC is a complex and highly heterogeneous tumour, involving multiple anatomical sites, which poses significant challenges for treatment. Despite therapeutic interventions, over half of HNSCC patients experience local or distant recurrence. For patients with recurrent or metastatic (R/M) HNSCC, the median OS ranges from 10 to 13 months. In this context, the development of novel therapies with enhanced efficacy and reduced adverse effects is urgently needed to improve patient survival and quality of life.

Our previous studies demonstrated that CTSL is significantly overexpressed in poorly differentiated HNSCC and that its expression positively correlates with cervical lymph node metastasis.^32^ Furthermore, we elucidated that CTSL promotes laryngeal cancer progression through the IL6‐JAK‐STAT3 signalling pathway.^7^ Additionally, other studies have reported that excessive accumulation of CTSL in intracellular and extracellular spaces regulates downstream targets and protein cascades associated with various malignant cells, impacting cancer cell apoptosis, angiogenesis and metastasis.^33^ In basal‐like breast cancer, inhibition of KDM4C promotes the recruitment of CTSL to chromatin through the transcription factor GRHL2. Methylation of GRHL2 at lysine 453 activates the histone‐cleaving activity of CTSL. Knockout of CTSL restores the tumour‐suppressive function lost upon KDM4C depletion.^34^ Moreover, CTSL is abundantly expressed in both tumour and stromal compartments of invasive breast cancer tissues.^35^ The specific CTSL inhibitor Z‐FY‐CHO may serve as a critical target for the treatment and early detection of metastatic HNSCC.

Emerging evidence suggests that DUBs regulate various biological and pathological processes by stabilizing tumour‐associated proteins. Several DUB inhibitors have been employed in tumour treatment models, underscoring their potential as therapeutic targets. As a member of the largest DUB subfamily, USP20 plays a pivotal role in various cancers by stabilizing oncogenic proteins. Studies have shown that USP20 recognizes and stabilizes hypoxia‐inducible factor 1α, thereby enhancing the transcription of hypoxia‐induced element genes.^36^ This, in turn, activates multiple signalling pathways involved in cancer progression, including angiogenesis, cell survival and invasion.^37^, ^38^ Additionally, USP20 modulates diverse biological processes by stabilizing both oncogenic and tumour‐suppressive proteins. Research indicates that USP20 triggers the activation of multiple pathways, including Wnt, MAPK, HIF1, NF‐κB, cell cycle checkpoints and other signalling cascades,^39^, ^40^, ^41^ thereby promoting the progression of various cancer types. In this study, the USP20‐CTSL axis was identified as a molecular marker and potential therapeutic target for metastatic HNSCC.

Recent studies have further revealed that USP20 can promote bladder cancer progression by regulating the Hippo‐YAP1 signalling pathway. USP20 directly interacts with YAP1 and inhibits its K48‐linked polyubiquitination, thereby enhancing the expression of YAP1 and its downstream target genes.^42^ In T‐cell acute lymphoblastic leukaemia (T‐ALL), USP20 has been identified as a super‐enhancer‐regulated driver gene that promotes tumour cell survival by deubiquitinating and stabilizing HIF1A. The USP20 inhibitor GSK2643943A exhibits significant anti‐tumour activity.^43^ Through multiple mechanisms, USP20 maintains the activity of oncogenic signalling pathways and participates in tumour cell hypoxia adaptation, proliferation and invasion. In this study, the USP20‐CTSL axis was identified as a molecular marker and potential therapeutic target for metastatic HNSCC, providing new insights for the development of USP20‐targeted therapies.

Ubiquitination and deubiquitination are opposing post‐translational modifications that fine‐tune protein stability, localization and activity. E3 ubiquitin ligases and DUBs are key modulators of cellular homeostasis and are frequently dysregulated in cancer.^44^, ^45^ Previous studies have identified STUB1 as a functional E3 ligase that mediates the ubiquitination and degradation of various oncogenic proteins.^22^, ^46^, ^47^, ^48^ Here, we report a novel regulatory axis wherein USP20, a DUB, competes with STUB1 to modulate CTSL expression and EMT progression in HNSCC.

Mechanistically, our data suggest that USP20 interacts with CTSL and promotes its expression by preventing STUB1‐mediated ubiquitination and degradation. Co‐immunoprecipitation assays revealed that USP20 and STUB1 competitively bind to CTSL, and overexpression of USP20 reduces CTSL ubiquitination levels. Interestingly, the suppression of STUB1 enhances CTSL stability, phenocopying the effect of USP20 overexpression. These findings imply a competitive regulatory model in which USP20 antagonizes STUB1 activity to maintain high levels of CTSL, thereby facilitating EMT. Given that CTSL has been associated with aggressive tumour phenotypes and poor prognosis in various cancers,^49^ our study suggests that the USP20‐STUB1‐CTSL axis may serve as a potential biomarker and therapeutic target in HNSCC. Furthermore, we found that knockdown of USP20 or inhibition of CTSL reversed EMT phenotypes, including downregulation of N‐cadherin and vimentin and restoration of E‐cadherin, highlighting the functional importance of this pathway in EMT induction. Previous work has linked CTSL to antigen processing and immunosuppression,^50^ and whether USP20 contributes to immune evasion via CTSL regulation warrants further investigation.

In addition, while our study suggests that USP20 may play a role in enhancing chemosensitivity, particularly to cisplatin and paclitaxel, further validation is required to confirm the therapeutic relevance of this finding. The limited evidence for drug sensitivity in our current model, remains a significant gap that requires additional experimental work. This could further solidify the utility of USP20 as a potential therapeutic target, not only in EMT regulation but also in overcoming chemoresistance in HNSCC.

In conclusion, our study reveals a novel mechanism by which USP20 promotes EMT and potentially metastasis in HNSCC through competitive inhibition of STUB1‐mediated CTSL degradation. These findings not only advance our understanding of EMT regulation but also underscore the therapeutic value of targeting the USP20‐CTSL axis in epithelial cancers.

## AUTHOR CONTRIBUTIONS

Lunhua Guo, Baihui Zhang and Xiaoqiao Cui jointly completed the writing and experimental work of this paper. Xueying Wang and Jiaqing Xiao participated in part of the experiments. Ji Sun, Kaibin Song and Susheng Miao supervised and guided the entire process and also provided financial support.

## CONFLICT OF INTEREST STATEMENT

The authors declare that they have no known competing financial interests or personal relationships that could have appeared to influence the work reported in this paper

## ETHICS STATEMENT

The patient specimens used in this article have been approved by the Ethics Committee of Harbin Medical University.

## Supporting information



Supporting Information
Table S1 All oligonucleotides sequences
Table S2 USP20 levels and clinicopathological features in 78 HNSCC patients.
Table S3 Prognostic factors in HNSCC cancer patients by univariate analysis.
Table S4 Multivariate analysis using the Cox proportional hazards model.
**Figure S1. CTSL expression and regulatory mechanisms in HNSCC cells. A‐B** Western blot analysis of CTSL protein levels following stable knockdown of CTSL using three independent shRNAs compared with negative control in FaDu (A) and HN8 (B) cells. **C** Cells were transfected with control shRNA (shNC) or shCTSL and treated with or without the AKT activator Sc‐79 (10 µM). Protein levels of CTSL, phosphorylated AKT (p‐AKT), total AKT, E‐cadherin (E‐ca) and N‐cadherin (N‐ca) were detected by immunoblotting, with β‐tubulin as the loading control. **D** Cells transfected with control vector or CTSL‐overexpression plasmid (OE‐CTSL) were treated with or without the AKT inhibitor MK2206 (2 µM). Expression of CTSL, p‐AKT, AKT, E‐ca and N‐ca was determined by immunoblotting, with β‐tubulin as the loading control. **E‐F** Quantification of CTSL protein levels over time following treatment with the proteasome inhibitor MG132 (5 µM) or the lysosomal inhibitor chloroquine (CQ, 25 µM) in FaDu (E) and HN8 (F) cells. Data are presented as mean ± SD (n = 3). **G** Schematic diagram of the workflow for immunoprecipitation (IP) and mass spectrometry (LC‐MS/MS) analysis of CTSL‐interacting proteins in FaDu cells. **H** Schematic network showing major deubiquitinases identified as CTSL‐interacting proteins, with USP20 being the most abundant. **I‐J** Flag‐CTSL, HA‐USP14(I) or HA‐USP12(J) was transfected into HEK293T cells for 48 hours. **K** Schematic representation of the structure of CTSL. **L** A plasmid encoding full‐length Flag‐CTSL was co‐transfected into 293T cells with full‐length (FL) HA‐USP20 or USP20 deletion mutant plasmids (Zf‐UBP, UCH and DUSP). Cell lysates were immunoprecipitated using anti‐HA antibody and subsequently immunoblotted with anti‐Flag antibody. **M** qPCR analysis of CTSL mRNA levels in FaDu cells after USP20 knockdown. Data are presented as mean ± SD (n = 3). Statistical differences were assessed using one‐way ANOVA. ns, not significant.
**Figure S2. Effects of Diclofenac on CTSL and USP20 Expression and Ubiquitination. A** 293T cells were treated with diclofenac for 0, 6 and 9 h. CTSL and USP20 levels were analysed by Western blotting **B** Western blot analysis of MG132‐treated CTSL and control cells with or without Diclofenac, showing levels of CTSL. **C** Western blot analysis of HN8 and Fadu cells treated with cycloheximide (CHX) and Diclofenac for 0, 6, 12, or 24 hours, assessing protein stability of HN8 and Fadu. **D** IP of HA‐Ub in MG132‐treated CTSL cells with Diclofenac treatment for 0, 6, or 9 hours, followed by Western blot for CTSL ubiquitination (Anti‐Ub) and CTSL levels. **E** Western blot analysis of HA‐USP20 and CTSL cells treated with Diclofenac for 0, 6, 9, 12, or 15 hours, showing levels of USP20, CTSL. **F** Western blot analysis of HA‐USP20‐WT and HA‐USP20‐CA in CTSL cells treated with Diclofenac for 0, 6, 9, 12, or 15 hours, showing levels of USP20 and CTSL. **G** Western blot analysis of CTSL cells transfected with si‐NC or si‐USP20 and treated with Diclofenac, showing levels of USP20 and CTSL. **H** qPCR analysis of CTSL mRNA levels in FaDu cells after STUB1 knockdown. **I‐J** Western blot analysis showing the knockdown efficiency of USP20 in HN8 cells using three different shRNAs (shUSP20#1–#3) compared with the negative control (shNC) and the overexpression of USP20 (USP20‐OE) compared with the control group (CTRL) in Fadu cells.
**Figure S3. USP20 and CTSL influence cell migration and proliferation in FaDu cells. A** Western blot analysis of USP20 and CTSL expression in FaDu cells. Cells were transfected with control or USP20 knockout and treated with or without CTSL overexpression. **B** Wound healing assay in FaDu cells at 0h and 36h following the indicated treatments. The graph on the right quantifies the wound healing percentage at 36h. **C** Representative images of colony formation assay and quantification of total colony counts in FaDu cells under the indicated conditions. Data are presented as mean ± SD (n = 3). Statistical differences were assessed using one‐way ANOVA. *p < 0.05, **p < 0.01, ***p < 0.001.

## Data Availability

The datasets used and/or analysed during the current study are available from the corresponding author on reasonable request.
