# Supplementary material for: USP20 competitively binds to STUB1 to enhance CTSL expression and promote epithelial‐mesenchymal transition in head and neck squamous cell carcinoma
Source: Clin Transl Med. 2025 Nov 19;15(11):e70520. doi: 10.1002/ctm2.70520 (PMC12629859; doi:10.1002/ctm2.70520)
Supplement: Supplementary file 1 — Supporting Information Table S1 All oligonucleotides sequences Table S2 USP20 levels and clinicopathological features in 78 HNSCC patients. Table S3 Prognostic factors in HNSCC cancer patients by univariate analysis. Table S4 Multivariate analysis using the Cox proportional hazards model. Figure S1. CTSL expression and regulatory mechanisms in HNSCC cells. A‐B Western blot analysis of CTSL protein levels following stable knockdown of CTSL using three independent shRNAs compared with negative control in FaDu (A) and HN8 (B) cells. C Cells were transfected with control shRNA (shNC) or shCTSL and treated with or without the AKT activator Sc‐79 (10 µM). Protein levels of CTSL, phosphorylated AKT (p‐AKT), total AKT, E‐cadherin (E‐ca) and N‐cadherin (N‐ca) were detected by immunoblotting, with β‐tubulin as the loading control. D Cells transfected with control vector or CTSL‐overexpression plasmid (OE‐CTSL) were treated with or without the AKT inhibitor MK2206 (2 µM). Expression of CTSL, p‐AKT, AKT, E‐ca and N‐ca was determined by immunoblotting, with β‐tubulin as the loading control. E‐F Quantification of CTSL protein levels over time following treatment with the proteasome inhibitor MG132 (5 µM) or the lysosomal inhibitor chloroquine (CQ, 25 µM) in FaDu (E) and HN8 (F) cells. Data are presented as mean ± SD (n = 3). G Schematic diagram of the workflow for immunoprecipitation (IP) and mass spectrometry (LC‐MS/MS) analysis of CTSL‐interacting proteins in FaDu cells. H Schematic network showing major deubiquitinases identified as CTSL‐interacting proteins, with USP20 being the most abundant. I‐J Flag‐CTSL, HA‐USP14(I) or HA‐USP12(J) was transfected into HEK293T cells for 48 hours. K Schematic representation of the structure of CTSL. L A plasmid encoding full‐length Flag‐CTSL was co‐transfected into 293T cells with full‐length (FL) HA‐USP20 or USP20 deletion mutant plasmids (Zf‐UBP, UCH and DUSP). Cell lysates were immunoprecipitated using anti‐HA antibody and subsequently i [file CTM2-15-e70520-s001.docx]

| Type | | Target | | | Sequence(5’-3’) | |
| --- | --- | --- | --- | --- | --- | --- |
| Plasmids | |  | | |  | |
| sh#1USP20 | | H-USP20 | | CGACACCTTCATCAAGTTGAA | |  |
| sh#2USP20 | | H-USP20 | | GCCCATCAGAAGATGAGTTCT | |  |
| sh#3USP20 | | H-USP20 | | CTATGTTGGCTGCGGAGAATC | |  |
| sh#1CTSL | | H-CTSL | | TGCCTCAGCTACTCTAACATT | |  |
| sh#2CTSL | | H-CTSL | | GAATTGCCTCAGCTACTCTAA | |  |
| sh#3CTSL | | H-CTSL | | AGGCGATGCACAACAGATTAT | |  |
| sgSTUB1#1 | | H-STUB1 | | CGAGGAACGGCGCATCCACC | |  |
| sgSTUB1#2 | | H-STUB1 | | ACTTGCGGCCCACGAAGAGC | |  |
| siRNA | |  | |  | |  |
| si-USP20#1 | | H-USP20 | | GCCAGAACGUGAUCAAUGG | |  |
| si-USP20#2 | | H-USP20 | | GGACAAUGAUGCUCACCUA | |  |
| si-USP20#3 | | H-USP20 | | GCGAGUGGCUCAACAAGUU | |  |
| Primers | |  | | |  | |
| Forward | | H-CTSL | | AAACTGGGAGGCTTATCTCACT | |  |
| Reverse | | H-CTSL | | GCATAATCCATTAGGCCACCAT | |  |
| Forward | | H-GAPDH | | ACAACTTTGGTATCGTGGAAGG | |  |
| Reverse | | H-GAPDH | | GCCATCACGCCACAGTTTC | |  |

Table S1 All oligonucleotides sequences

| Characteristic | Total | USP20 | | P |
| --- | --- | --- | --- | --- |
|  |  | Low | High |  |
| Total | 78 | 33 | 45 |  |
| Age |  |  |  | 0.169 |
| <60 | 37 | 19 | 18 |  |
| ≥60 | 41 | 14 | 27 |  |
| Gender |  |  |  | 0.159 |
| Male | 62 | 29 | 33 |  |
| Female | 16 | 4 | 12 |  |
| Tumor Size |  |  |  | 0.015 |
| < 4cm | 59 | 20 | 39 |  |
| ≥4cm | 19 | 13 | 6 |  |
| Lymph node metastasis |  |  |  | <0.001 |
| Negative | 49 | 30 | 19 |  |
| Positive | 29 | 3 | 26 |  |
| KI67 expression |  |  |  | 0.492 |
| Low | 38 | 18 | 20 |  |
| High | 40 | 15 | 25 |  |
| Differentiation |  |  |  | 0.653 |
| Well | 40 | 18 | 22 |  |
| Poor | 38 | 15 | 23 |  |
| Vascular invasion |  |  |  | 0.246 |
| Negative | 45 | 22 | 23 |  |
| Positive | 33 | 11 | 22 |  |
| AJCC stage |  |  |  | <0.001 |
| I/Ⅱ | 42 | 28 | 14 |  |
| Ш/IV | 36 | 5 | 31 |  |

Table S2 USP20 levels and clinicopathological features in 78 HNSCC patients.

| Parameter | n | cumulative survival rates(%) | | Mean survival  time(mo) | Hazard ratio | 95% Confidence  interval | P value |
| --- | --- | --- | --- | --- | --- | --- | --- |
|  | 78 | 3-years | 5-years |  |  |  |  |
| Age |  |  |  |  | 0.712 | 0.298-1.702 | 0.445 |
| <60 | 37 | 78.8 | 64.4 | 37.9 |  |  |  |
| >60 | 41 | 72.7 | 62.3 | 28.2 |  |  |  |
| Gender |  |  |  |  | 2.17 | 0.819-5.747 | 0.119 |
| Male | 62 | 82.5 | 72.6 | 36.8 |  |  |  |
| Female | 16 | 59.7 | 51.1 | 17.4 |  |  |  |
| Tumor Size |  |  |  |  | 0.4 | 0.132-1.212 | 0.105 |
| <4cm | 59 | 69.1 | 54.4 | 27.1 |  |  |  |
| >4cm | 19 | 82.1 | 70.4 | 50.7 |  |  |  |
| Lymph node metastasis |  |  |  |  | 13.294 | 3.634-48.636 | ＜0.001 |
| Negative | 49 | 93.4 | 85.7 | 68.9 |  |  |  |
| Positive | 29 | 50.6 | 44.3 | 6.3 |  |  |  |
| KI67 expression |  |  |  |  | 0.623 | 0.260-1.492 | 0.288 |
| Low | 38 | 78.1 | 62.8 | 34.4 |  |  |  |
| High | 40 | 73.6 | 64.4 | 31.3 |  |  |  |
| Differentiation |  |  |  |  | 0.66 | 0.276-1.579 | 0.35 |
| Well | 40 | 74.2 | 63.5 | 33 |  |  |  |
| Poor | 38 | 72.8 | 63.7 | 32.6 |  |  |  |
| Vascular invasion |  |  |  |  | 1.061 | 0.452-2.495 | 0.891 |
| Negative | 45 | 81.8 | 68.9 | 36.7 |  |  |  |
| Positive | 33 | 68.2 | 56.9 | 27.5 |  |  |  |
| AJCC stage |  |  |  |  | 2.404 | 0.925-6.249 | 0.072 |
| I/ⅡI | 42 | 82.7 | 75.6 | 52.3 |  |  |  |
| Ш/IV | 36 | 64 | 57.6 | 10 |  |  |  |
| USP14 |  |  |  |  | 217.99 | 1.795-26478.33 | 0.028 |
| Low | 33 | 96.6 | 88.9 | 71.4 |  |  |  |
| High | 45 | 81.7 | 27.6 | 4.5 |  |  |  |
| CTSL |  |  |  |  | 5.985 | 2.242-15.974 | ＜0.001 |
| Low | 34 | 90.2 | 85.9 | 53.3 |  |  |  |
| High | 44 | 87.1 | 32.1 | 16.7 |  |  |  |

Table S3 Prognostic factors in HNSCC cancer patients by univariate analysis.

| Parameter | n | Hazard ratio | 95% Confidence | P value |
| --- | --- | --- | --- | --- |
|  |  |  | interval |  |
| Age |  | 3.298 | 0.749-14.519 | 0.114 |
| <60 | 35 |  |  |  |
| 60 | 32 |  |  |  |
| Gender |  | 0.619 | 0.152-2.525 | 0.503 |
| Male | 51 |  |  |  |
| Female | 16 |  |  |  |
| Tumor Size |  | 0.362 | 0.059-2.212 | 0.271 |
| <4cm | 52 |  |  |  |
| >4cm | 15 |  |  |  |
| Lymph node metastasis |  | 5.425 | 0.711-41.41 | 0.103 |
| Negative | 40 |  |  |  |
| Positive | 27 |  |  |  |
| KI67 expression |  | 0.14 | 0.017-1.158 | 0.068 |
| Low | 36 |  |  |  |
| High | 31 |  |  |  |
| Differentiation |  | 1.491 | 0.414-5.375 | 0.541 |
| Well | 31 |  |  |  |
| Poor | 36 |  |  |  |
| Vascular invasion |  | 0.253 | 0.048-1.351 | 0.108 |
| Negative | 40 |  |  |  |
| Positive | 27 |  |  |  |
| AJCC stage |  | 4.009 | 0.441-36.463 | 0.218 |
| I/II | 37 |  |  |  |
| II/IV | 30 |  |  |  |
| USP20 |  | 149.178 | 5.195-4283.734 | 0.003 |
| Low | 31 |  |  |  |
| High | 36 |  |  |  |
| CTSL |  | 11.436 | 0.946-138.204 | 0.055 |
| Low | 33 |  |  |  |
| High | 34 |  |  |  |

Table S4 Multivariate analysis using the Cox proportional hazards model.


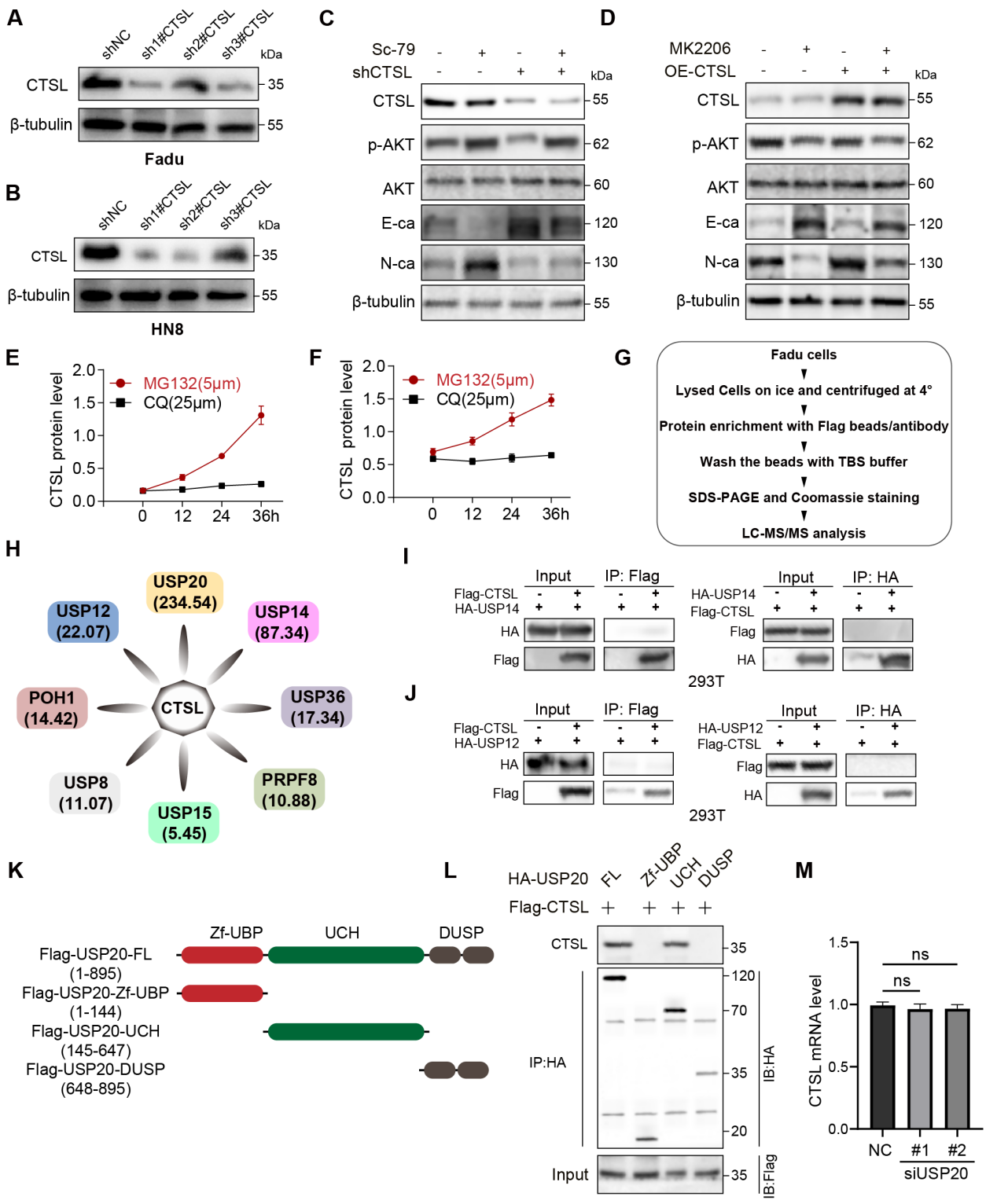


**Fig S1. CTSL expression and regulatory mechanisms in HNSCC cells. A-B** Western blot analysis of CTSL protein levels following stable knockdown of CTSL using three independent shRNAs compared with negative control in FaDu (A) and HN8 (B) cells. **C** Cells were transfected with control shRNA (shNC) or shCTSL and treated with or without the AKT activator Sc-79 (10 μM). Protein levels of CTSL, phosphorylated AKT (p-AKT), total AKT, E-cadherin (E-ca), and N-cadherin (N-ca) were detected by immunoblotting, with β-tubulin as the loading control. **D** Cells transfected with control vector or CTSL-overexpression plasmid (OE-CTSL) were treated with or without the AKT inhibitor MK2206 (2 μM). Expression of CTSL, p-AKT, AKT, E-ca, and N-ca was determined by immunoblotting, with β-tubulin as the loading control. **E-F** Quantification of CTSL protein levels over time following treatment with the proteasome inhibitor MG132 (5 μM) or the lysosomal inhibitor chloroquine (CQ, 25 μM) in FaDu (E) and HN8 (F) cells. Data are presented as mean ± SD (n = 3). **G** Schematic diagram of the workflow for immunoprecipitation (IP) and mass spectrometry (LC-MS/MS) analysis of CTSL-interacting proteins in FaDu cells. **H** Schematic network showing major deubiquitinases identified as CTSL-interacting proteins, with USP20 being the most abundant. **I-J** Flag-CTSL, HA-USP14(I) or HA-USP12(J) was transfected into HEK293T cells for 48 hours. **K** Schematic representation of the structure of CTSL. **L** A plasmid encoding full-length Flag-CTSL was co-transfected into 293T cells with full-length (FL) HA-USP20 or USP20 deletion mutant plasmids (Zf-UBP, UCH, and DUSP). Cell lysates were immunoprecipitated using anti-HA antibody and subsequently immunoblotted with anti-Flag antibody. **M** qPCR analysis of CTSL mRNA levels in FaDu cells after USP20 knockdown. Data are presented as mean ± SD (n = 3). Statistical differences were assessed using one-way ANOVA. ns, not significant.


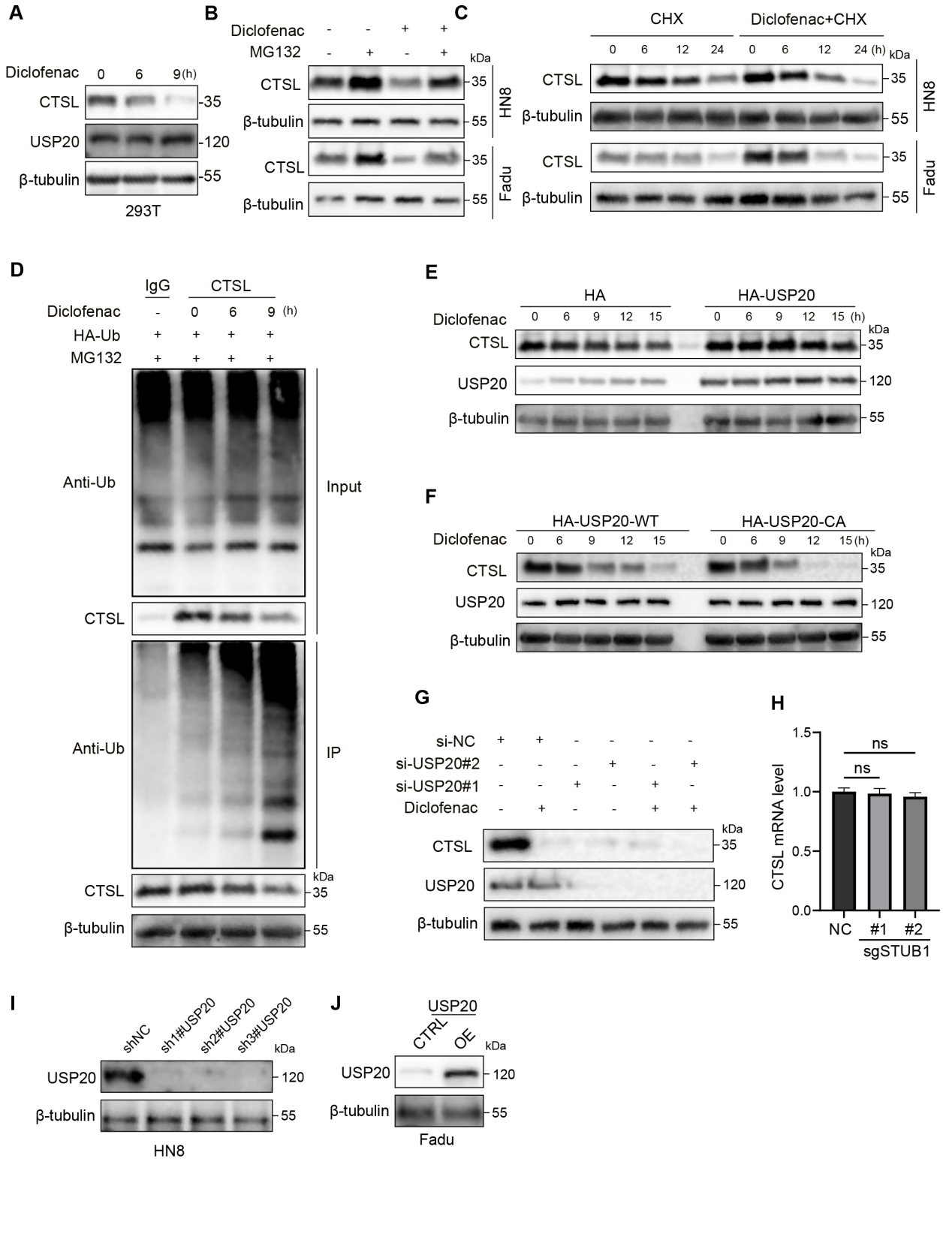


**Fig S2. Effects of Diclofenac on CTSL and USP20 Expression and Ubiquitination. A** 293T cells were treated with diclofenac for 0, 6, and 9 h. CTSL and USP20 levels were analyzed by Western blotting **B** Western blot analysis of MG132-treated CTSL and control cells with or without Diclofenac, showing levels of CTSL. **C** Western blot analysis of HN8 and Fadu cells treated with cycloheximide (CHX) and Diclofenac for 0, 6, 12, or 24 hours, assessing protein stability of HN8 and Fadu. **D** IP of HA-Ub in MG132-treated CTSL cells with Diclofenac treatment for 0, 6, or 9 hours, followed by Western blot for CTSL ubiquitination (Anti-Ub) and CTSL levels. **E** Western blot analysis of HA-USP20 and CTSL cells treated with Diclofenac for 0, 6, 9, 12, or 15 hours, showing levels of USP20, CTSL. **F** Western blot analysis of HA-USP20-WT and HA-USP20-CA in CTSL cells treated with Diclofenac for 0, 6, 9, 12, or 15 hours, showing levels of USP20 and CTSL. **G** Western blot analysis of CTSL cells transfected with si-NC or si-USP20 and treated with Diclofenac, showing levels of USP20 and CTSL. **H** qPCR analysis of CTSL mRNA levels in FaDu cells after STUB1 knockdown. **I-J** Western blot analysis showing the knockdown efficiency of USP20 in HN8 cells using three different shRNAs (shUSP20#1–#3) compared with the negative control (shNC) and the overexpression of USP20 (USP20-OE) compared with the control group (CTRL) in Fadu cells.


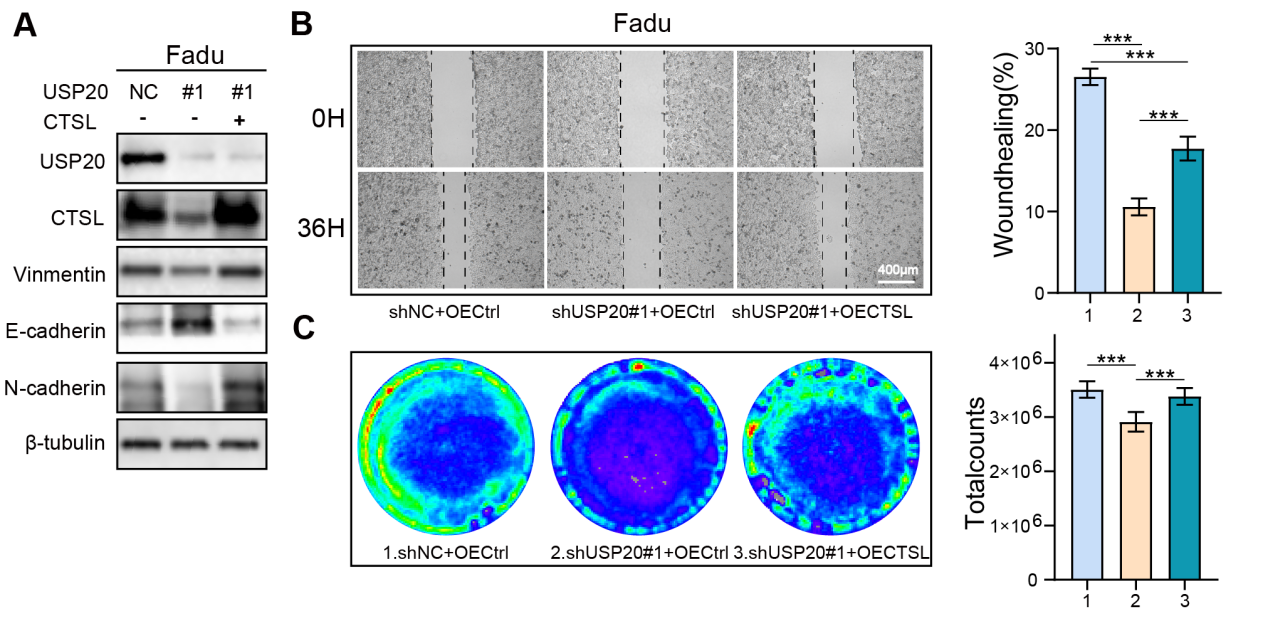


**Fig S3. USP20 and CTSL influence cell migration and proliferation in FaDu cells. A** Western blot analysis of USP20 and CTSL expression in FaDu cells. Cells were transfected with control or USP20 knockout and treated with or without CTSL overexpression. **B** Wound healing assay in FaDu cells at 0h and 36h following the indicated treatments. The graph on the right quantifies the wound healing percentage at 36h. **C** Representative images of colony formation assay and quantification of total colony counts in FaDu cells under the indicated conditions. Data are presented as mean ± SD (n = 3). Statistical differences were assessed using one-way ANOVA. *p < 0.05, **p < 0.01, ***p < 0.001.
